# How low can you go? Establishing detection limits for rare eukaryotes in Southern Ocean sedimentary ancient DNA

**DOI:** 10.1093/bioadv/vbag113

**Published:** 2026-06-05

**Authors:** E Elisa Davis, Jane Younger, Christopher Burridge, Linda Armbrecht

**Affiliations:** Institute for Marine and Antarctic Studies (IMAS), Ecology and Biodiversity Centre, University of Tasmania, Hobart, TAS 7004, Australia; Australian Centre for Excellence in Antarctic Science, IMAS, University of Tasmania, Hobart, TAS 7004, Australia; Institute for Marine and Antarctic Studies (IMAS), Ecology and Biodiversity Centre, University of Tasmania, Hobart, TAS 7004, Australia; School of Natural Sciences, University of Tasmania, Hobart, TAS 7004, Australia; Institute for Marine and Antarctic Studies (IMAS), Ecology and Biodiversity Centre, University of Tasmania, Hobart, TAS 7004, Australia; Australian Centre for Excellence in Antarctic Science, IMAS, University of Tasmania, Hobart, TAS 7004, Australia

## Abstract

**Motivation:**

Sedimentary ancient DNA (*sed*aDNA) is genetic material extracted from paleoarchives. It provides insights into the composition and dynamics of ecosystems over time. Such information can be crucial in anticipating how ecological communities may respond to environmental shifts within the context of the current climate crisis. However, challenges exist in accurately verifying ancient DNA from ecologically significant vertebrate species (e.g. fishes, aquatic birds, and mammals). These species occur only in trace amounts in sedimentary records. Here, we benchmark a stringent bioinformatic pipeline using synthetic and empirical metagenomic *sed*aDNA data from IODP Expedition 382 (Scotia Sea). Our objectives are threefold: (i) test taxonomic assignment precision for rare marine eukaryotes, (ii) evaluate taxonomic assignment sensitivity across different sediment ages, and (iii) establish the minimum sequence quantity necessary for robust identification.

**Results:**

We demonstrate that taxonomic assignment precision varied significantly with sequence quantity and metagenomic context. Assignment sensitivity decreased with taxonomic rank and database representation. Reliable detection of low-abundance taxa in *sed*aDNA is achievable with 250 and 500 DNA fragments at the family and genus level, respectively. The reanalysis of IODP Exp. 382 *sed*aDNA data, using a custom built marine vertebrate-focused reference database, resulted in the first genetic reconstruction of the vertebrate community in the Scotia Sea. This lays the groundwork for future investigations into the presence and biodiversity of Southern Ocean vertebrates using *sed*aDNA.

**Availability and implementation:**

All project related scripts and generated simulated datasets are available in ae_fishing_benchmark repository (https://github.com/33davis/ae_fishing_benchmark). The demultiplexed raw data in relation to the IODP Exp. 382 U1538 reanalysed during this study is available in the NCBI Sequence Read Archive database (https://www.ncbi.nlm.nih.gov/sra) under Accession code/BioProject PRJNA861836 (BioSamples SAMN29928044 - SAMN29928123) and includes metadata for each sediment and control sample.

## 1 Introduction

Sedimentary ancient DNA (*sed*aDNA) is genetic material from dead organisms preserved in sediments (Parducci *et al.* 2019, Capo *et al.* 2022). Analysis of *sed*aDNA provides insights into the long-term dynamics of aquatic and terrestrial ecosystems. It also helps reveal community composition in response to climate variation (e.g. De Schepper *et al.* 2019, Armbrecht *et al.* 2022, Kjær *et al.* 2022, Zimmermann et al. 2023). Recent technical advancements have improved marine *sed*aDNA coring and sample collection (Armbrecht *et al.* 2020). In addition, DNA extraction methods (Armbrecht *et al.* 2020, Heintzman *et al.* 2023), sequencing (Key *et al.* 2017, Hu *et al.* 2021), and bioinformatic tools, pipelines, and databases have advanced (Schubert *et al.* 2014, Huson *et al.* 2016, Murchie *et al.* 2021, Collin *et al.* 2020, Fellows Yates *et al.* 2021, Pochon *et al.* 2023). For example, bioinformatic tools can authenticate ancient DNA (Hübler *et al.* 2019), remove modern contaminants (Huson *et al.* 2016), and improve taxonomic assignments at finer resolution (Vogel *et al.* 2023).

Despite recent improvements in *sed*aDNA techniques, challenges remain in identifying ‘rare’ taxa, which, in *sed*aDNA records, encompass eukaryotic organisms, as they represent minute proportions compared to prokaryotes (Capo *et al.* 2022, Wood *et al.* 2025). For example, a shotgun metagenomic analysis of *sed*aDNA from samples collected offshore Tasmania, Australia, revealed that only 1.37% of the sequencing reads were of eukaryotic origin, as identified using the 18S rRNA taxonomic marker gene (Armbrecht *et al.* 2021). To overcome these limitations and gain deeper insights into specific eukaryotes, metabarcoding (amplification of certain gene regions using primers) or hybridization capture (amplification of stretches of DNA using complementary DNA or RNA probes) can be useful (Murchie *et al.* 2021, Armbrecht *et al.* 2021, 2024). Species of interest may be ecologically significant sentinel species (whales, seals, seabirds, sharks), mesopredators (fishes, cnidaria), and keystone taxa such as krill (Younger *et al.* 2016, McCormack *et al.* 2021, Bista *et al.* 2023). Their rare representation in *sed*aDNA is likely due to patchy preservation of their DNA relative to that of ubiquitous planktonic microorganisms (Huston *et al*. 2023).

In the Southern Ocean, contemporary declines in Patagonian and Antarctic toothfish and emperor penguins have been reported in response to climate-change-induced decreases in prey availability and sea ice loss (Griffiths *et al.* 2017, Trathan *et al.* 2020). Studies of Antarctic lake sediments and historic terrestrial breeding grounds have highlighted the potential of *sed*aDNA research to demonstrate changes in marine predator populations over the past century to 5 kya (Zhou *et al.* 2024, Wood *et al.* 2025). However, deeper time (100 kya and beyond) investigations into sentinel species have rarely been attempted, owing to the challenges for eukaryotes highlighted above, but are important for better understanding how they have responded to past climate change.

Here, we assess the performance of current *sed*aDNA data-processing techniques for detecting eukaryotes (in particular, Southern Ocean vertebrates), which are rare relative to prokaryotes in metagenomic datasets. Building upon the observed challenges and the significance of rare taxa detection, we evaluate the reliability of taxonomic assignments across varying numbers of input reads, levels of *sed*aDNA damage, and sample complexity. This study utilizes previously published metagenomic *sed*aDNA data from IODP Expedition 382 (Scotia Sea) and simulated *sed*aDNA datasets of rare Southern Ocean vertebrates (penguin and icefish) to benchmark an established bioinformatic workflow.

## 2 Methods

### 2.1 Bioinformatics overview and dataset description

**Figure 1:**
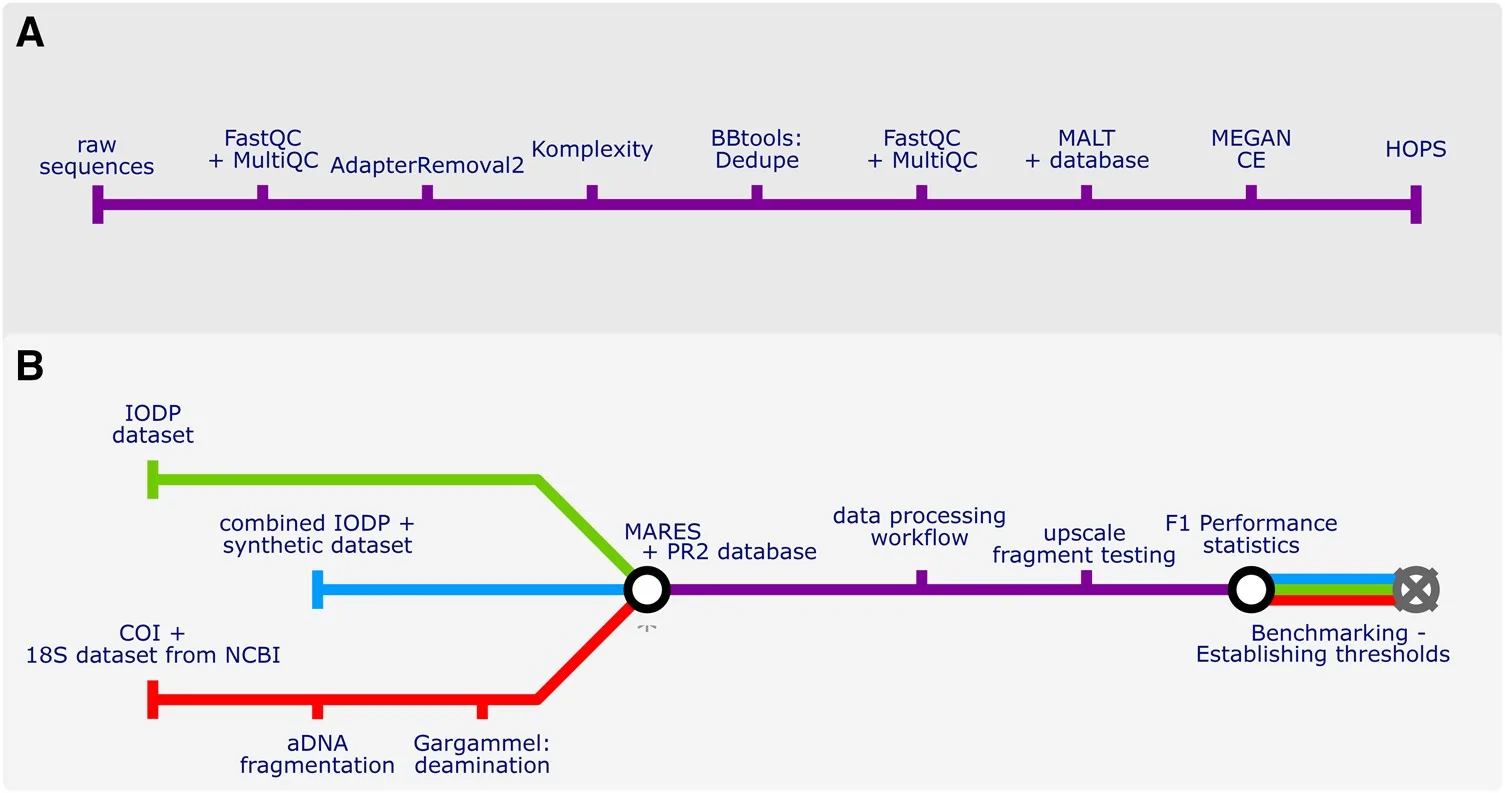
Overview of the bioinformatic pipeline and input datasets. (A) *sed*aDNA data processing steps from raw sequencing data, quality control (FastQC + MultiQC), filtering (Komplexity, Dedupe), mapping to a database and visualization [MALT, MEGAN Community Edition (CE)], and ancient DNA damage assessment (HOPS). (B) Overview of the three different datasets used as input into the pipeline and statistical testing. We created the figure with Metro Designer (https://tennessine.co.uk/metro/).

We followed the stringent, established *sed*aDNA bioinformatic pipeline described in Armbrecht *et al.* (2020) to process three different datasets (see below; Fig. 1A; Supplementary Information Methods 1, available as supplementary data at *Bioinformatics Advances* online).

### 2.2 Datasets

#### 2.2.1 IODP expedition 382 *sed*aDNA dataset

The metagenomic shotgun data used in this study originated from sediment cores collected during IODP Expedition 382, “Iceberg Alley and Sub-Antarctic Ice and Ocean Dynamics,” in March–May 2019. Details on coring locations, cores, *sed*aDNA-clean sampling procedures, and the age model can be found in Weber *et al.* (2021) and Armbrecht *et al.* (2022). Our study reanalysed *sed*aDNA data from Site U1538 (Pirie Basin, central Scotia Sea, 3130 m water depth). This is a densely sampled core with 33 samples. The recovered genetic material dates back to 417 kya (Armbrecht *et al.* 2022). We re-processed the sample raw reads and eight control samples [one air and one drill fluid control, and six extraction blank controls (EBCs)], following the steps in Supplementary Methods 1.1, available as supplementary data at *Bioinformatics Advances* online, for a total of 41 samples. We retained all metagenomic reads from the samples, except contaminant reads (found in controls), which we removed from the sample data.

#### 2.2.2 IODP expedition 382 *sed*aDNA dataset

We created synthetic datasets featuring Southern Ocean sentinel species. For 10 Notothenioidei (icefish) and 10 Sphenisciformes (penguins) species, we included Cytochrome Oxidase I (COI) barcode gene sequences. For chinstrap penguin (*Pygoscelis antarcticus*) and Antarctic spiny plunderfish (*Harpagifer antarcticus*), we also included 18S rRNA gene sequences (Supplementary Table 1, available as supplementary data at *Bioinformatics Advances* online). The sequences were downloaded from the National Center for Biotechnology Information GenBank nucleotide database on 30 January 2024 (https://www.ncbi.nlm.nih.gov/nucleotide/).

We simulated ancient DNA fragmentation by first deriving a probability density function from the fragment length distribution averaged across all IODP Exp. 382 core U1538 *sed*aDNA samples (Supplementary Fig. 1, available as supplementary data at *Bioinformatics Advances* online). Using a Monte Carlo algorithm (Supplementary Methods 2, available as supplementary data at *Bioinformatics Advances* online; https://github.com/33davis/ae_fishing_benchmark), we iteratively fragmented each sentinel barcode sequence according to this distribution to generate stochastic fragment variation. We then repeated this sampling to generate per-sequence sets containing ***n ***= 10, 25, 50, 100, 250, 500, 1000, 2500, and 5000 synthetic ancient-sized fragments, minimizing any bias from uniform fragment lengths in downstream processing. As with DNA fragmentation, we also simulated different degrees of DNA damage to reflect empirical damage patterns. We chose three U1538 core samples: U1538_1H_1 (core top, 0 m below seafloor—0 kya), U1538_2H_6 (middle, 14.55 mbsf—14.5 kya), and U1538_8H_2 (bottom, 61.35 mbsf—214 kya) that exhibited 3% (top), 7% (middle), and 18% (bottom) eukaryote *sed*aDNA damage (Armbrecht *et al.* 2022). Thus, we randomly subsampled 3%, 7%, 18% (and for comparison also 0% and 100%) of the synthetic DNA fragments and damaged (deaminated) them using the deamSim program of Gargammel (https://github.com/grenaud/gargammel; Renaud *et al.* 2017). We used the Briggs *et al.* (2007) parameters for modelling ancient DNA deamination: nick frequency = 0.0024, length overhanging reads = 0.36, probability of deamination for double-stranded reads = 0.0097, and probability of deamination for single-stranded reads = 0.68, then converted the resulting fasta files to fastq format using seqtk. Then we merged the damaged and undamaged fragments (seqtk seq -F '#' in.fa > out.fq; https://github.com/lh3/seqtk), resulting in synthetic datasets with varying degrees of damage and input DNA fragment numbers *n*. Finally, we combined the IODP Exp. 382 + synthetic datasets to create a complex metagenomic sample context. In summary, we processed 41 samples from the IODP Exp. 382 U1538 core, 45 synthetic datasets, and 35 combined synthetic and IODP datasets.

### 2.3 Database construction and alignments

We combined two publicly available alignment databases: the Protist Ribosomal Reference (PR2) database (Guillou *et al.* 2013) and the Marine Eukaryote Species (MARES) Cytochrome Oxidase I (COI) database (Arranz *et al.* 2020). This combination allows comparisons of the vertebrate-seeded IODP data (MARES) while maintaining a complex protist background (PR2) typical of marine *sed*aDNA datasets. For details on database construction, see Supplementary Methods 3, available as supplementary data at *Bioinformatics Advances* online. All datasets were aligned against the combined database using MALT (v.0.61), and the resulting blastn files were converted to .rma6 files, imported into MEGAN Community Edition (v.6.25.6; Huson *et al.* 2016). Inside MEGAN CE, we subtracted any taxa identified in the controls from the sample data. As an exploratory check for preferential assignment between COI and 18S barcode genes using the MARES + PR2 database, a Kruskal-Wallis rank-sum test was performed post-alignment. We compared percent identity scores between the different gene assignments (COI, 18S, and 16S), with post-hoc pairwise comparisons using Dunn’s test (and Benjamini-Hochberg adjusted *P* values).

### 2.4 Taxonomic data analysis

#### 2.4.1 Positive predictive value (PPV; precision), recall (sensitivity), and F1 score

We utilized binary classifier metrics, positive predictive value (PPV, also known as precision) and Recall (also known as sensitivity), to assess the performance of our pipeline in detecting Southern Ocean vertebrate taxa. These classifiers are robust when working with imbalanced datasets (Saito and Rehmsmeier 2015), e.g. when differential taxonomic preservation and presence are present, as in *sed*aDNA. Sensitivity is the proportion of true positives correctly assigned (here, the detection of rare vertebrates at each taxonomic level). Precision is defined as the proportion of positive assignments that are true positives (here, the proportion of classifications assigned to the correct taxonomic level, such as genus or family). Furthermore, we included the F1 metric used in previous metagenomic benchmarking studies to assess overall pipeline performance (Wood *et al.* 2019, Burks *et al.* 2023, Pusadkar and Azad 2023). We calculated precision and sensitivity at the species, genus, and family levels for each dataset.


$$\mathrm{Sensitivity}=\frac{\#\;\mathrm{correct}\;\mathrm{read}\;\mathrm{assignments}}{\mathrm{total}\;\#\;\mathrm{of}\;\mathrm{known}\;\mathrm{reads}}$$

$$\mathrm{Precision}=\frac{\#\;\mathrm{correct}\;\mathrm{read}\;\mathrm{assignments}}{\left(\#\;\mathrm{correct}\;\mathrm{read}\;\mathrm{assign}.+\#\;\mathrm{incorrect}\;\mathrm{read}\;\mathrm{assign}.\right)}$$

$$F1\;\mathrm{Score}=2*\frac{\left(\mathrm{Precision}*\mathrm{Sensitivity}\right)}{\left(\mathrm{Precision}+\mathrm{Sensitivity}\right)}$$

#### 2.4.2 Statistical analysis

Precision, sensitivity, and F1 (aka metrics) were bounded between 0 and 1 and displayed non-normal, heteroscedastic distributions. Therefore, we employed nonparametric approaches, in particular Spearman correlations (Williams 1986), to test for continuous monotonic trends in fragment complexity (***n***), damage treatment, performance metric type, and sample context. We define sample context as either simple, consisting only of synthetic sequences with no metagenomic background, or complex, combining synthetic sequences with IODP metagenomic core samples.

To assess potential overfitting of the tested variables, we applied Kruskal-Wallis rank-sum tests across relevant strata (e.g. damage treatments, sample context, and rank combinations) to determine whether metric distributions differed significantly. If no significant differences were found within the tested grouping (*P* > .05), we aggregated our data (averaging metric values at each *n*) for that variable. Where differences were significant, data were analysed separately. This approach ensured aggregation was data-driven and validated across multiple experimental factors rather than assumed (Supplementary Methods 4, available as supplementary data at *Bioinformatics Advances* online).

Based on Spearman correlations, we determined the lowest fragment quantity***n*** at which pipeline performance significantly improved. We set three criteria to be fulfilled for determining the lowest quantity threshold: (i) Spearman correlation rho value was positive, indicating a positive monotonic trend between the performance metric and quantity value, (ii) rho was statistically significant (*P* value <.05) after Benjamini-Hochberg correction for multiple tests, and (iii) observed performance metric value was greater than 0.99. All statistical analyses were conducted using R (version 4.5.1).

## 3 Results

### 3.1 IODP Exp. 382 site U1538 taxonomic composition

Mapping the Exp. 382 U1538 shogun data against the MARES + PR2 database assigned a total of 122,132 reads across all samples. In 32 out of the 33 core samples, reads were assigned to eukaryotes, with the majority being from two samples: U1538C_2H_4 (28 822 reads) and U1538C_2H_5 (20 193 reads). In U1538C_7H_6, no reads were assigned to eukaryotes; however, this sample already had low read counts prior to mapping (0.1 M reads).

**Figure 2:**
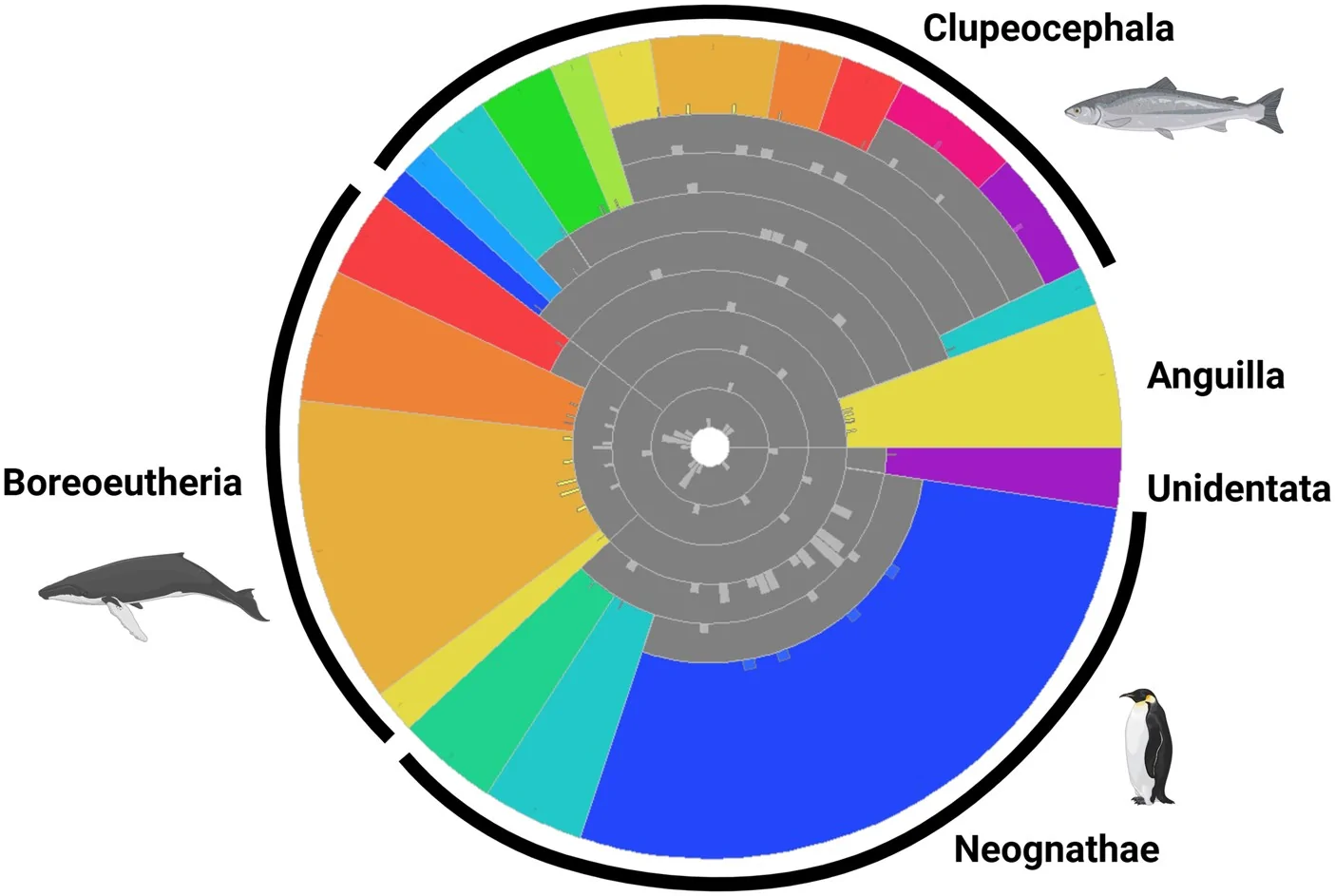
Vertebrata composition and abundance at IODP Exp. 382 Site U1538. Light grey bars indicate per-sample assignment counts, and radial sections represent nested taxonomic assignments. Detailed vertebrate assignment records are supplied in Supplementary File 4, available as supplementary data at *Bioinformatics Advances* online. All coloured sections indicate a family-level assignment, and all grey sections indicate sub-class or higher-level taxonomic assignment, increasing in level towards the centre ring (Vertebrata). Black bars outside the radial graph indicate the broad taxonomic grouping of assignments in that section. The figure was generated in MEGAN CE v. 6.25 and Biorender.

Across all 33 samples, 114 reads were assigned to the subphylum Vertebrata (Fig. 2), representing ∼0.09% of all assigned reads (average of 3.65 Vertebrata reads per sample). Read lengths of these were 31–132 bp (average 64.6 bp ± 22.2 bp). They mapped to marker genes 18S rRNA (53.8%), COI (43.6%), and 16S rRNA (2.6%), respectively. The Kruskal-Wallis rank-sum test comparing percent identity scores across assignments to different genes (COI, 18S, and 16S) indicated significant differences between reads mapped to 18S rRNA and COI genes (adjusted *P* value = 1.42e-07) and between reads mapped to COI and 16S rRNA genes (adjusted *P* value = .0387).

Within Vertebrata, infraclass Neognathae was the most frequently detected group, with a total of 21 reads assigned in seven samples (max. of four reads in sample 7H_4: 54 mbsf—188 kya). Neognathae were followed by the clade Euteleostomi, with 16 fragments assigned across 11 samples. A total of 31 (Vertebrata) fragments mapped to the species level within the orders Teleostei, Boreouthea, and Sauria (in descending order). The average length of assigned reads was 62.3 ± 21.7 bp. There were no discernible trends in sample depth, and no significant differences in assignment to different genes; however, we noted a higher proportion of reads mapped to the COI (65%) than to the 18S rRNA gene, and a single assignment to the 16S rRNA gene. More detailed information on these assignments is supplied in the Supplementary Results 1, available as supplementary data at *Bioinformatics Advances* online.

We detected *sed*aDNA damage across samples, with a general increase in percent damage with depth (Supplementary File 6, available as supplementary data at *Bioinformatics Advances* online). We measured 0% *sed*aDNA damage in four samples: 1H_1 (0 mbsf—0 kya), 7H_6 (57.55 mbsf—203 kya), 8H_3 (62.85 mbsf—215 kya), and 14H_1 (118.35—392 kya). For samples 1H_1, 7H_6, and 8H_3, no reads passed through stringent filtering criteria or matched to taxa in our custom database. In contrast, in sample 14H_1, three reads passed through filtering and assignment but did not show *sed*aDNA damage. However, in our study, maximum damage was found in sample 7H_2 (51.85 mbsf—177 kya). After HOPS damage analysis, only three rare vertebrate hits were characterized as ancient, all attributed to Delphinidae at 1.4 kya (1.45 mbsf), 74 kya (29.55 mbsf), and 126 kya (41.69 mbsf).

### 3.2 Synthetic dataset taxonomic composition and pipeline performance

Across datasets with varying levels of damage (0%, 3%, 7%, 18%, and 100%), fragments were consistently assigned at different taxonomic levels (e.g. cellular organisms vs. species). Damage analysis (via HOPS) failed for all synthetic datasets with***n ***< 50 (as anticipated, since this input (***n***) was below the minimum required for HOPS). In all other synthetic datasets (***n ***> 50), *sed*aDNA damage was observed and consistent with the applied degree of damage (Supplementary File 6, available as supplementary data at *Bioinformatics Advances* online).

**Figure 3:**
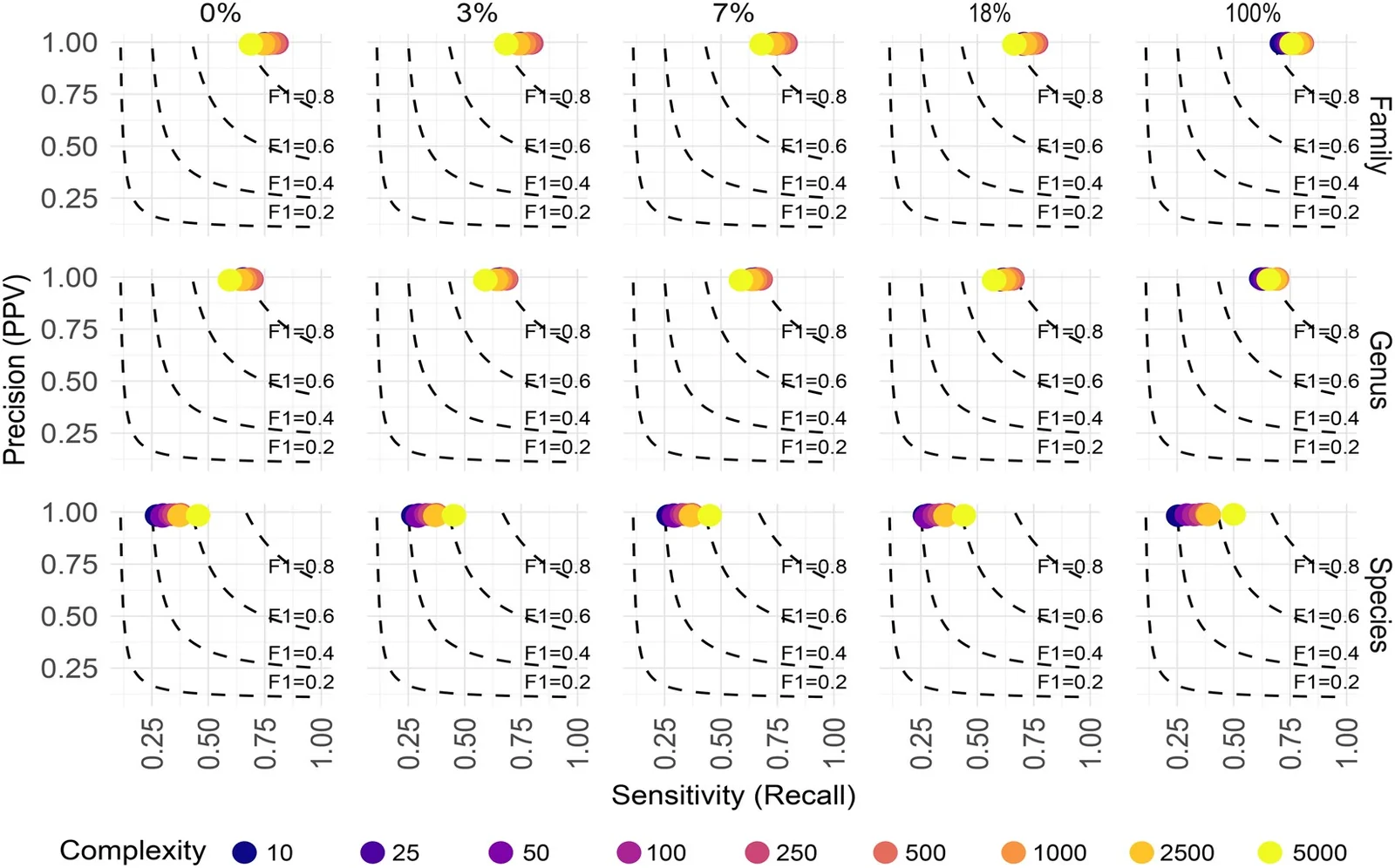
Precision (positive predictive value) versus sensitivity (recall) in taxonomic assignments using the 45 synthetic datasets post alignment to the MARES + PR2 database. Panels show different damage treatments (columns) and taxonomic ranks (rows). Dotted contour lines visualize F1 scores, where F1 = 0.5 corresponds to performance equal to random chance. The synthetic datasets represented by each damage treatment, including their corresponding depths and ages, are described in Section 2.2.2. An overview of the performance statistics is provided in Supplementary File 2, available as supplementary data at *Bioinformatics Advances* online.

Precision remained consistently high (near 1.0) across all synthetic datasets, regardless of input fragment quantity***n*** or damage level applied (Fig. 3). In contrast, sensitivity varied consistently between taxonomic resolutions, with the lowest sensitivity values observed at the species level. Therefore, even with the simplest synthetic dataset (***n*** = 10, damage = 0%), the pipeline’s ability to detect rare vertebrates decreases as taxonomic resolution becomes finer. Pairwise Kruskal-Wallis tests and a generalized linear model (Supplementary File 7 and Supplementary Results 2.2, available as supplementary data at *Bioinformatics Advances* online) indicated that sample context and taxonomic rank were significant interaction terms for fragment quantity, but damage treatment was not. Spearman correlations across all simple context datasets revealed no significant global monotonic read thresholds (passing our three-set criteria); we cannot establish minimum fragment thresholds based solely on synthetic datasets. (Supplementary File 7, available as supplementary data at *Bioinformatics Advances* online). Species-level sensitivity of taxonomic assignments varies depending on the species (Supplementary Fig. 4, available as supplementary data at *Bioinformatics Advances* online). Fragments from blackfin notothen, *Trematomus scotti* (COI gene—OK493634), showed the highest sensitivity across all datasets and species. Across all datasets, at least one fragment was correctly assigned at the species level. For the two species for which we had included both COI and 18S rRNA gene sequences, *H. antarcticus* (Antarctic spiny plunderfish) and *Pygoscelis antarcticus* (chinstrap penguin), species-specific assignment occurred only with 18S fragments (*H. antarcticus*) or with COI fragments (*P. antarcticus*; Supplementary Figs 5–7, available as supplementary data at *Bioinformatics Advances* online).

**Figure 4:**
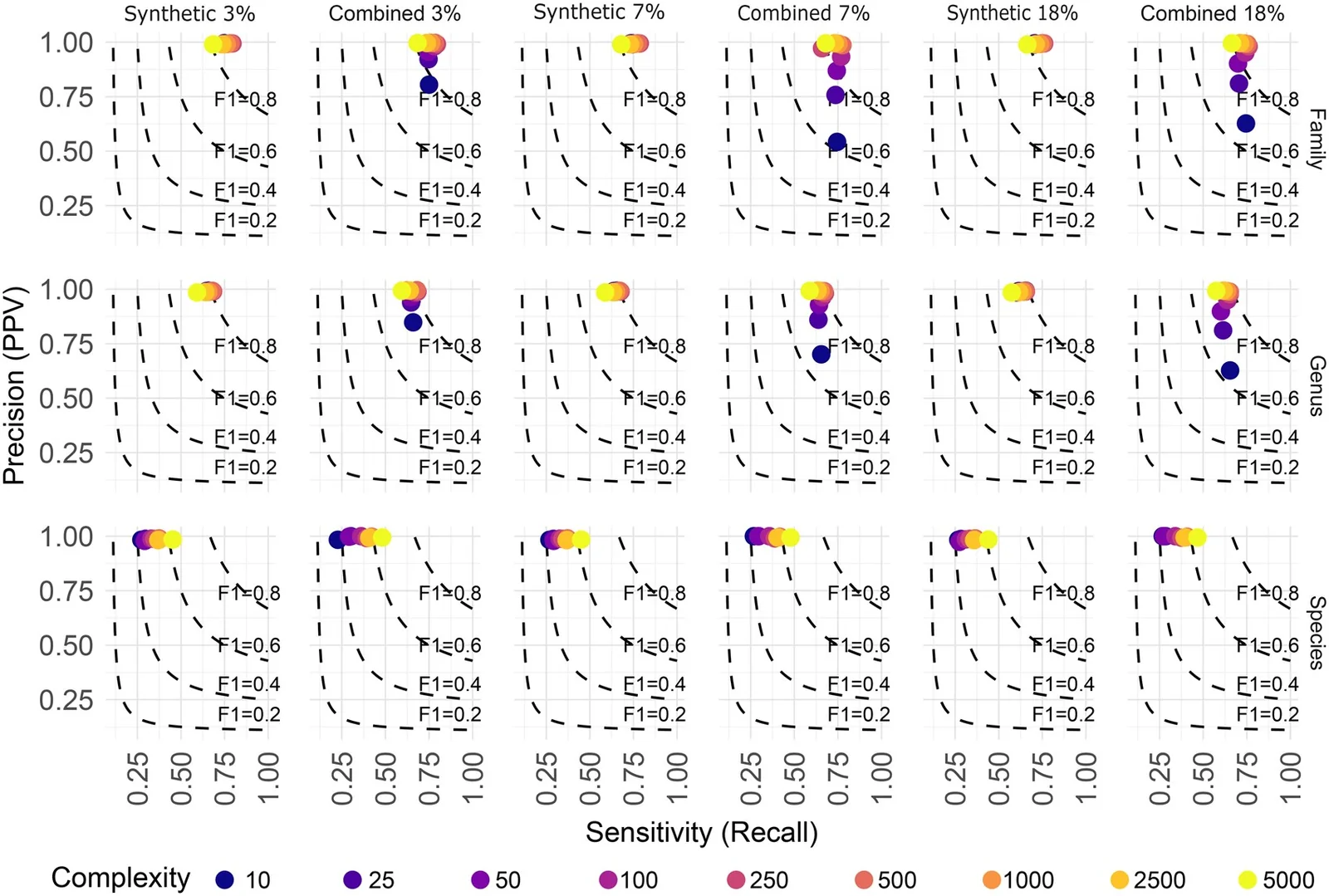
Precision (positive predictive value) versus sensitivity (recall) in taxonomic assignments comparing 35 synthetic datasets (damage treatments 3%, 7%, and 18%) and the same 35 synthetic datasets merged with IODP Exp. 382 U1536C samples, post alignment to the MARES + PR2 database. Panels show different damage treatments and dataset types (columns: synthetic and combined) and taxonomic ranks (rows). Dotted contour lines visualize F1 scores, where F1 = 0.5 corresponds to performance equal to random chance. An overview of the performance statistics is provided in Supplementary File 2, available as supplementary data at *Bioinformatics Advances* online.

### 3.3 Combined (IODP Exp. 382 U1528 + synthetic) dataset taxonomic composition and pipeline performance

Rare taxa were detected across all input***n*** quantities. We observed misassignments in sample 8H_2 (***n ***= 5000), where two COI fragments (accession OK493624) originating from *Artedidraco lonnbergi* were identified as belonging to the family Bathydraconidae. At lower input fragment quantities (***n ***≤ 2500), fragments from this species were correctly assigned to the family Artedraconidae or to the suborder Notheniodei.

As synthetic datasets with an increasing amount of***n*** fragments were combined with IODP samples, the calculated percent *sed*aDNA damage values for the combined ‘samples’ were lower than those for the corresponding reanalysed IODP samples, as the inclusion of synthetic fragments with IODP reads made the relative proportion of possibly ancient reads determined by HOPS smaller. The *sed*aDNA damage values of the combined datasets were: 1H_1 (3% ± 2%), 2H_6 (5% ± 3%), and 8H_2 (10% ± 6%) assignments of the combined IODP + synthetic datasets reflected the results of the synthetic datasets and IODP Exp. 382 data.

When comparing synthetic and combined dataset performance, the most pronounced change was a decrease in combined dataset precision at low input fragment quantities (10, 25, 50 fragments) at the genus and family ranks (Fig. 4). This reflects an increase in false-positive assignments in highly imbalanced datasets. Despite this, precision at the family level remained higher than at genus or species levels. F1 contour lines showed that the best overall performance—balancing precision and sensitivity—was at the family level in moderately damaged combined datasets. Precision at the species level did not decline; however, sensitivity was markedly lower, indicating a decrease in species detection. While sensitivity did not vary significantly (Fig. 4), precision showed a significant positive monotonic trend with increasing input fragment quantity (***n***) (i.e. a strong positive correlation between increasing numbers of rare vertebrate DNA fragments and their correct assignments; Table 1). Our three criteria for determining the lowest required fragment quantity threshold were met twice: at the family level, precision approached the peak pipeline performance value of 1 with a significantly positive trend at 250 fragments per rare vertebrate, while 500 fragments were required at the genus level. If we lower our metric cut-off criteria from 0.99 to 0.95 (increasing the chance that each rare vertebrate assignment has a 5% probability of being incorrect), the minimum fragment quantity threshold substantially decreased to only 50 fragments for both family and genus level assignments.

**Table 1:** Minimum fragment quality thresholds.^a^

Taxonomic rank	Input fragment quantity	Rho at threshold	*P* value (BH)	Metric value	Metric cutoff (>)	Expected error
Family	250	0.9208244	0.37E−10	0.9907591	0.99	1%
Genus	500	0.9060912	2.38E−10	0.9955042	0.99	1%
Family	50	0.9208244	0.37E−10	0.9549689	0.95	5%
Genus	50	0.9060912	2.38E−10	0.9638336	0.95	5%
Family	25	0.9208244	0.37E−10	0.9218750	0.90	10%
Genus	25	0.9060912	2.38E−10	0.9390244	0.90	10%

a The thresholds are derived from Spearman correlations of monotonic relationships between precision metric and all tested variables, filtered by strict metric criteria stated in Supplementary Methods 3.2, available as supplementary data at *Bioinformatics Advances* online. *P* values shown are post Bejamani-Hochberg (BH) corrections for multiple comparisons. The full results of calculated Spearman correlations can be found in Supplementary File 8, available as supplementary data at *Bioinformatics Advances* online.

## 4 Discussion

### 4.1 Key findings and conclusions

Our study benchmarked a stringent bioinformatic pipeline for detecting and authenticating low-abundance taxa in complex metagenomic *sed*aDNA samples. Previous bioinformatic benchmarking studies have primarily focused on optimizing metagenomic data processing for automation, reproducibility, or classifier performance (Fellows Yates *et al.* 2021, Pochon *et al.* 2023, Pusadkar and Azad 2023) or solving specific ancient DNA-related problems such as ancient damage toolkit development, benchmarking ancient DNA-aware quality control software, or ancient environmental pangenome mapping (Michelsen *et al.* 2022, Lien *et al.* 2023, Vogel *et al.* 2023). However, to date, no study has explicitly addressed or tested the precision and sensitivity of taxonomic assignments of low-abundance taxa. We validated rare taxa identification using empirical and synthetic *sed*aDNA data with modelled deamination, fragmentation, and a range of input fragment quantities. Our established minimum fragment thresholds (family = 250, genus = 500) were significantly positively correlated with high taxonomic assignment precision. Overall, our study demonstrated that low-abundance vertebrate taxa can be detected and identified in metagenomic samples from ancient environments.

### 4.2 Sensitivity and precision analysis

To enable an evaluation of pipeline performance using realistic ancient metagenomic data, we merged our synthetic datasets of rare vertebrate sequences with an existing, empirical IODP Exp. 382 U1528 *sed*aDNA dataset that included microbial and eukaryotic sequences. This allowed us to evaluate how rare eukaryotic taxa are detected among complex, diverse *sed*aDNA signals. Even in simple synthetic datasets, we observed an underlying trend: sensitivity (proportion of correct assignments) decreased as taxonomic resolution increased. Sensitivity was highly species-dependent at the finest taxonomic resolution, suggesting that reference bias toward organisms well represented in the database likely played a role (Chorlton 2024). This is also supported by the observed differential level of taxonomic assignment for the same species when mapping the 18S vs. COI synthetic fragments without additional complexity of IODP samples (Supplementary Figs 4–7, available as supplementary data at *Bioinformatics Advances* online). These findings showed that taxonomic classification at the family level is most appropriate for ecological studies that utilize curated barcode gene databases, such as MARES and PR2. This aligns with Northern Hemisphere marine *sed*aDNA studies, which often adopt family-level resolution due to stringent filtering or limited genus-level assignments (Djurhuus *et al.* 2020, Zimmerman *et al.* 2023, Kjær *et al.* 2022). Our study provides quantitative support for this practice in the context of rare eukaryotic taxa.

Overall, Spearman correlation trends indicated that to achieve near-optimal rare-taxa assignment with our pipeline, at least 250 assigned fragments per family are required to achieve near-total confidence in rare-taxon presence in a *sed*aDNA sample. A lower input fragment quantity threshold could be accepted, but the chances of false positive assignments increase. In our synthetic dataset analysis, precision was the only metric that showed a significant monotonic trend with the quantity of fragment input. Unexpectedly, the combination of neither precision nor sensitivity with fragment quantity was significantly affected by deamination. Pusadkar and Azad (2023) conducted a benchmarking study using the F1 score to evaluate the taxonomic performance of metagenomic profilers on simulated ancient microbial community sequencing data with various combinations of deamination and contamination (human and microbial). They found that deamination alone had a minimal effect on F1 scores and overall taxonomic assignments. Similarly, sensitivity was relatively low, but precision remained high (Pusadkar and Azad 2023), consistent with our results for the simple synthetic datasets. However, we are aware that only limited damage treatments were tested in our benchmarking study, which also aimed to address the effects of low and high input fragment quantities, leading to a complex study design (80 synthetic datasets + 41 IODP Exp. 382 core samples). Future research could focus on using synthetic datasets with the same number of input sequences (>250) and varying deamination levels to elucidate the precision-sensitivity-damage relationship more clearly.

F1 scores can provide general pipeline performance information and are primarily used to compare taxonomic classification models with imbalanced datasets (Wood *et al.* 2019). As the F1 score is the harmonic mean of precision and sensitivity, we found that any significant trends in precision or sensitivity would be reflected in the F1 score, without providing any further meaningful interpretation. Therefore, we focused on reporting precision, sensitivity, and the F1 score. Future studies utilizing the one-to-one definition for binary classifiers—e.g. the sensitivity of each fragment—would be integral to more powerful statistical validation of rare taxonomic assignments in metagenomic *sed*aDNA samples (Saito and Rehmsmeier 2015). Along with increased statistical power, utilizing the one-to-one classifier definition would allow for the incorporation of testable variables that affect rare taxonomic assignments, such as the effects of database bias and contamination.

### 4.3 Expedition 382 *sed*aDNA reanalysis for Southern ocean vertebrates and ecological implications

Compared to Armbrecht *et al.* (2022), the overall eukaryotic assignments decreased by 41% at IODP Exp. 382 Site U1538, which can be attributed to the selection of a different reference database, namely PR2 + MARES. The latter included COI marine eukaryote reference sequences (Arranz *et al.* 2020), thus reads were mapped against a database with a smaller representation of species, whereas Armbrecht *et al.* (2022) focused on broad eukaryote screening (18S and 28S rRNA). In contrast to general eukaryotic assignments, vertebrate assignments increased twofold compared to the former study. Our damage analysis demonstrates most clearly that the average proportion of eukaryote *sed*aDNA damage was over double (36.15%) that reported in the previous study at site U1538 (an average of 11% ± 7%). Consistent with the original studies’ trend, our measured proportion of eukaryote *sed*aDNA damage steadily increased with sediment depth (Armbrecht *et al.* 2022). A recent Antarctic terrestrial *sed*aDNA study reported elevated levels of fragmentation and deamination damage in penguin DNA extracted from sediments, likely due to prolonged breakdown and interactions between large animals with sediment microbiome (Wood *et al.* 2025). Therefore, our elevated eukaryotic *sed*aDNA values indicate an overall increase in vertebrate assignment in IODP reanalysis.

We detected sequences of avian, fish, and mammal origin at Site U1538, marking the first marine *sed*aDNA record of marine megafauna in the Southern Ocean. We expected any vertebrate detections in the core to originate from ecological groups with large population biomass near the seafloor, such as benthic fish (Huston et al. 2023). Instead, Site U1538 has a diverse vertebrate representation interspersed throughout the core, with largest representation of hits coming from birds (Fig. 2). While this is in direct defiance of our expectations, it confirms previous predictions of long-term marine vertebrate presence and productivity in the Scotia Sea (Fordyce 1989, Younger *et al.* 2016, Bista *et al.* 2023). Finer taxonomic resolution of these hits cannot be verified; however, the read counts for these groups did not meet our confidence thresholds for precise assignment established during benchmarking. Although detailed ecological interpretation is limited, our results indicate the presence of major marine vertebrate groups at Site U1538. Therefore, the reanalysis of the U1538 samples as part of our benchmarking of the *sed*aDNA bioinformatic pipeline has been an important step towards validating Holocene-Pleistocene rare eukaryotic marine taxa in sediment cores. Furthermore, the development of a targeted bioinformatic pipeline or application of hybridization-capture methods for ancient marine megafauna could inform future work utilizing *sed*aDNA to elucidate how multiple trophic levels (primary producers, primary consumers, and apex predators) respond to longer-term climatic changes in a critical Southern Ocean region.

### 4.4 Methodological insights and future directions

We integrated the MARES and PR2 databases to achieve a balance between broad eukaryotic coverage and specificity for marine eukaryotes. While MARES is a curated metabarcoding database with the highest recorded proportion of marine taxa (Arranz *et al.* 2020), we observed several unexpected terrestrial vertebrate COI gene assignments in the IODP Exp. 382 U1538 reanalysis. Although these hits fell well below our established thresholds for confident assignment, their presence underscores our rationale for benchmarking established pipelines for rare eukaryote identification in *sed*aDNA. Broadly speaking, these outlier terrestrial classifications are often due to taxa underrepresentation in the database, contamination, or a combination of both. In our synthetic dataset tests, database underrepresentation of the tested icefish and penguin species contributed to the absence of correct species-level assignments and to variable taxonomic sensitivity among the tested taxa (Supplementary Figs 4–7, available as supplementary data at *Bioinformatics Advances* online). Arranz *et al.* (2020) justified the inclusion of many terrestrial species as representatives of related marine taxa with no current genetic records. While utilizing the MARES database did increase the vertebrate assignments in our reanalysis of Exp. 382 core U1538, terrestrial species assignments detract from any meaningful ecological interpretations or paleo-community inferences (Capo *et al.* 2022). Also, as our study does not explicitly test for database contamination, it cannot be ruled out as a contributing factor to our terrestrial species assignments during the IODP Exp. 382 reanalysis.

Database contamination is becoming better documented as sequencing becomes more accessible (SD Chorlton, 2024). A recent benchmarking study of barcoding gene databases, including MARES, showed that database contamination was prevalent (both human contamination and chimeric sequence data) if there was only one criterion (rather than several) used for database validation (Claver *et al.* 2023). Indeed, while Pusadkar and Azad (2023) did not explicitly test for database contamination, their study demonstrated that both human and homologous bacterial contamination significantly reduced the performance of ancient metagenomic profilers. While this presents a very interesting opportunity for future work to model the effects of database contamination in *sed*aDNA analysis, it presently highlights the current limitations of metabarcoding reference databases in metagenomic *sed*aDNA studies. Taken together with our benchmarking results, these findings highlight the need for very large databases with at least two types of validation to mitigate contamination and bias, as demonstrated by successful validation of rare eukaryotes in Northern Hemisphere *sed*aDNA studies (Chorlton 2024, Kjær *et al.* 2022, Wood *et al.* 2025). Our results underscore the current gap in understanding how database selection, taxonomic bias, and contamination quantitatively affect rare eukaryotic assignment in *sed*aDNA analyses, but indicate that large, cosmopolitan databases and independent validation are important stopgaps for the accurate capture of rare eukaryotic taxa.

Our study represents the first benchmarking of a sequencing data processing pipeline for rare eukaryotic taxa in imbalanced, ancient metagenomic contexts. As such, we established a significant correlation between taxonomic precision and fragment quantities, and the global lowest thresholds for confident and precise assignment of rare eukaryotes in a complex metagenomic context typical of *sed*aDNA. In this work, we also reanalysed metagenomic *sed*aDNA data from IODP Exp. 382 sediment core U1538 and have noted the earliest coarse detection of marine mammals, fish, and seabirds in the Scotia Sea. While lacking high resolution, our reanalysis highlights the potential value of *sed*aDNA for reconstructing long-term marine vertebrate presence in the Southern Ocean. Future methodological and bioinformatic refinements will be essential to enhance the resolution of Southern Ocean *sed*aDNA rare vertebrate signals, enabling palaeoecological interpretations comparable to those achieved in Northern Hemisphere studies (Zimmerman et al. 2023, Wood *et al.* 2025). These efforts will also provide a foundation for quantitatively optimizing current ancient DNA data processing pipelines to reproducibly validate rare ancient eukaryotic taxa in marine *sed*aDNA.

## Supplementary Material

vbag113_Supplementary_Data

## Data Availability

The demultiplexed raw data in relation to the IODP Exp. 382 U1538 reanalysed during this study is available in the NCBI Sequence Read Archive database (https://www.ncbi.nlm.nih.gov/sra) under Accession code/BioProject PRJNA861836 (BioSamples SAMN29928044–SAMN29928123) and includes metadata for each sediment and control sample. All data scripts are available at the GitHub repository (https://github.com/33davis/ae_fishing_benchmark).
