# Supplementary material for: How low can you go? Establishing detection limits for rare eukaryotes in Southern Ocean sedimentary ancient DNA
Source: Bioinform Adv. 2026 Jun 5;6(1):vbag113. doi: 10.1093/bioadv/vbag113 (PMC13539354; doi:10.1093/bioadv/vbag113)
Supplement: Supplementary file 1 [file vbag113_supplementary_data.zip › AE_Supplementary Informtion.docx]

Supplementary Information

*sedaDNA Bioinformatic Pipeline benchmarking for rare marine eukaryotic taxa*

Authors: E. Elisa Davis, Jane Younger, Christopher Burridge, Linda Armbrecht

Supplementary Methods:

1.Bioinformatic Processing Pipeline

1.1 Sequence data filtering

The raw read *sed*aDNA shotgun data were demultiplexed, adapters were trimmed and reads collapsed using ADAPTERREMOVAL v.2.1.7-foss02916a (--adapterlist –barcodelist, --collapse; Schubert *et al.,* 2016). Next, we generated quality control reports of the samples with FastQC and MultiQC (Hanson, 2010; Ewels *et al.*, 2016). Then, low complexity sequences were removed using Komplexity (Clarke *et al.,* 2019) with a threshold cut-off of 0.55***.*** The dedupe program within BBMAP v. 37.36 (Bushnell, 2014) was used to remove duplicate sequences created during sequencing, and then we generated a final quality control report with FastQC and MultiQC.

1.2 Sequence mapping

We utilised MALT (v.0.61; Huson *et al.,* 2016) to align the sequences to a database (for details on the database, see Section 3). Initially we built a database index (malt-build), and then taxonomic assignments were made (malt-run, semiglobal alignment; Herbig *et al.,* 2016). We converted the resulting .blastn files to .rma6-format for visualization of results in MEGAN Community Edition (v.6.25.9; Huson *et al.,* 2016).

1.3 *sed*aDNA damage assessment

The post-alignment output files (.rma6 format) were processed using the HOPS tool (v. 0.35-1; Hübler *et al*., 2019) to assess the proportion of ancient reads within each sample from MALT processed data. ‘Percent damage’ is calculated by taking the ratio of reads assigned to ancient (i.e., reads characterized by deamination of bases near sequence terminus) relative to reads assigned to default (i.e., reads that pass the stringent filtering criteria but do not show damage signals). The ‘eukaryote percent damage’ proxy estimates ancient DNA damage for eukaryotes only, which is achieved by specifying a ‘taxa list’ – a textfile that contains the word “Eukaryota”.

2.1 Synthetic dataset species

**Table 1.** List of sequences downloaded from NCBI and included in the synthetic dataset.

| Species | Common name | Accession No. | Gene | Length (bp) |
| --- | --- | --- | --- | --- |
| *Dissostichus eleginoides* | Patagonian toothfish | MW829388 | COI | 675 |
| *Harpagifer antarcticus* | spiny plunderfish | ON891147 | COI | 664 |
| *Trematomus scotti* | blackfin notothen | OK493634 | COI | 652 |
| *Trematomus loennbergii* | deepwater notothen | OK493720 | COI | 652 |
| *Dissostichus mawsoni* | Antarctic toothfish | ON000293 | COI | 635 |
| *Harpagifer bispinis* | Magellan plunderfish | OL339430 | COI | 670 |
| *Harpagifer kerguelensis* | Kerguelen spiny plunderfish | ON891164 | COI | 664 |
| *Harpagifer georgianus* | South Georgia spiny plunderfish | ON891170 | COI | 664 |
| *Champsocephalus gunnari* | mackerel icefish | EF609319 | COI | 655 |
| *Artedidraco lonnbergi* | NA | OK493624 | COI | 652 |
| *Aptenodytes patagonicus* | king penguin | EU525303 | COI | 913 |
| *Aptenodytes forsteri* | emperor penguin | EU525299 | COI | 911 |
| *Eudyptes chrysocome* | western rockhopper penguin | FJ582589 | COI | 902 |
| *Pygoscelis antarcticus* | chinstrap penguin | EU525471 | COI | 899 |
| *Pygoscelis adeliae* | Adélie penguin | MK261839 | COI | 896 |
| *Eudyptes chrysolophus* | macaroni penguin | FJ582593 | COI | 848 |
| *Eudyptes filholi* | eastern rockhopper penguin | MG739962 | COI | 846 |
| *Eudyptes schlegeli* | royal penguin | MG740185 | COI | 846 |
| *Eudyptula minor* | little blue penguin | EU525360 | COI | 841 |
| *Megadyptes antipodes* | yellow-eyed penguin | MK262211 | COI | 842 |
| *Pygoscelis antarcticus* | chinstrap penguin | KP875235 | 18S | 1820 |
| *Harpagifer antarcticus* | Antarctic spiny plunderfish | AF518190 | 18S | 1582 |

2. 2 Synthetic datasets fragmentation

We created a personalised fragmentation script for our synthetic datasets, as our gene inputs could not utilised in built-in fragmentation algorithm (fragSim) from Gargammel (Renaud *et al*., 2019). For the fragment, we utilised the same principles as fragSim algorithm to generate fragments from a logarithmic distribution curve of possible fragment lengths. To derive logarithmic distribution of fragment lengths based on U1538 data, we extracted read length distributions of raw U1538 sample files generated from MULTIQC. In R, we log transformed fragment lengths to estimate the logarithmic mean (meanlog) and standard deviation of empirical samples. We then used the logarithmic mean and standard deviation fragment length values as required parameters for our scripts.

Supplementary Figure 1. Fragment length probability density distribution. Plot was generated in R with geometric mean = 3.999 and standard deviation = 0.19, derived from fragment lengths from U1538 site reanalyses.

A minimum (25 bp) and maximum fragment (100) cutoff value based on sequencing values (Armbrecht *et al*., 2019). The algorithm created fragments iteratively and therefore would begin at the start of every input gene, with a random fragment length value from the established distribution would be chosen, the fragment would be written into a new file, and then a new fragment would be generated from the cutoff point of the last generated fragment. After fragmentation, synthetic datasets were deaminated as described in the main text.

HOPS damage analysis was conducted to confirm deamination of synthetic fragments. HOPS MaltExtract failed for all synthetic samples *n* < 50. This was expected as this is below the minimum input requirements for HOPS. However, in all other synthetic datasets *seda*DNA damage was noted in samples, indicating that Gargammel deamination damage was successful.

3. Database construction

The PR^2^ database (<https://pr2-database.org/#about>) is comprised of 18S rRNA reference sequences. At present, it contains over 220,000 sequences, although it focuses primarily on protists, it also contains sequences from metazoan, fungi, and plants (Guillou *et al.,* 2013). For this study, PR^2^ version 5.0.0 was utilized. The MARES reference sequence database (version 16/01/2023, downloaded on 09/02/2024) includes all eukaryotic families known to have marine species from the World Registry of Marine Species, WoRMs (WoRMS Editorial Board, 2020; <https://osf.io/8rdqk/>). The database contains all available eukaryote COI barcode labelled taxa from both the Barcode of Life Database (BOLD; Ratnasingham & Herbet, 2007; <https://boldsystems.org/>) and NCBI Genbank repositories. The MARES database was downloaded in fasta format (Arranz *et al.*, 2022). Of the two possible MARES database options, the ‘MARES_BAR’ version was chosen, meaning taxa compiled in database included the ‘BARCODE’ tag during the compiling process. Arranz *et al.*, (2022) reports this as the higher quality database, as reference samples labelled with “BARCODE” are better annotated, although there is a smaller breadth of taxa.

4. Statistical Analysis

**S1. Data structure and terminology**

- **Input fragment quantity (*n*)**: {10, 25, 50, 100, 250, 500, 1000, 2500, 5000}.
- **Damage treatments (simple context)**: no_damage (0%), mudline_damage (3%), middle_damage (7%), bottom_damage (18%), all_damage (100%).
- **Combined context**: synthetic datasets merged with IODP U1538 samples at 3%, 7%, and 18% damage (“spiked_*”).
- **Metrics**: precision (PPV), sensitivity (recall), and F1.

**S2. Pre-processing and long-format assembly**
We reshaped Supplementary Files 2–3 into a tidy format with variables: rank (Species, Genus, Family), metric (precision, sensitivity, F1), context (damage level or combined), context_type (simple vs combined), input_fragment (numeric), and metric_value.

**S3. Testing for aggregation feasibility**
To **check for overfitting and confirm whether aggregation was appropriate**, we applied **Kruskal–Wallis tests** across multiple grouping factors to determine whether metric distributions differed significantly. Tests included:

- Damage treatments within each *(rank × metric × context type)*
- Context differences within *(rank × metric)*
- Rank differences within *(context × metric)*
- Nested combinations (e.g., rank × context × metric)
  Where **p > 0.05**, we aggregated by averaging metric values across the tested grouping at each *n*. Otherwise, data remained separate. Outputs: kw_tests_by_context.csv.

**S4. Spearman correlations and BH correction**
We computed Spearman ρ between input_fragment and metric_value for:
(a) non-aggregated datasets (per context) and
(b) aggregated datasets (where KW indicated no significant differences).
BH correction was applied globally. Outputs: spearman_by_context.csv and spearman_aggregated.csv.

**S5. Threshold identification**
Minimum fragment quantity thresholds were flagged when all criteria were met: ρ > 0, BH-adjusted p < 0.05, and metric_value ≥ cutoff (0.99 primary; 0.95 and 0.90 relaxed). Results are in thresholds_*_aggregated.csv and thresholds_*_nonaggregated.csv.

**S6. Global LMM (descriptive)**
We fitted lmer(metric_value ~ input_fragment + context_type + (1|rank) + (1|context)) for precision to inspect fixed effects while accounting for random intercepts. Outputs: lmm1_anova_precision.csv.

**S7. Reproducibility**
All analyses were performed in R (v4.5.1) using tidyverse, readxl, rstatix, lme4, and lmerTest. Full code is provided in aggregation_text_file.txt.

Supplementary Results:

1.IODP Exp. 382 Vertebrate Species Assignments

31 fragments within Vertebrata are assigned at the species level, and of these only 9 fragments were assigned to ecologically likely Southern Ocean species. Seven of the 31 fragments were assigned to the common Atlantic bottlenose dolphin (*Tursiops truncatus)* in five samples. All seven fragments aligned to the same 18S sequence (NCBI accession number ABRN02390690.56.1590_U) were, on average, 46 ± 7.8 bp in length and had an average identity score of 93.7%. We queried the assigned reads against the NCBI nucleotide database (saved search 25.01.2025), which confirmed that they matched short mRNA and nuclear sequences of Cetacean species.

Two of the 31 fragments matched ecologically significant Southern Ocean species, specifically, fin whale (*Balaenoptera physalus*) (sample 8H1_1, 59.85 mbsf – 210 kya) and Ross seal (*Ommatophoca rossii*) (sample 4H_3, 29.55 mbsf – 74 kya) (Fig. 2). These two fragments were 80 and 76 bp long, respectively. When the two were re-queried against NCBI, the fin whale hit matched equally to different cetacean species, while the query of the Ross seal fragment revealed a 100% identity match to the Ross seal COI gene (Accession numbers: GU167277.1 and AY377132.1. The remaining 22 of 31 fragments were assigned at the species level, mapped to unlikely marine and terrestrial candidates for the Scotia Sea, ranging from aquaculture species *such as Salmo salar* (Atlantic salmon) to unexpected terrestrial species like *Aquila audax* (wedge-tailed eagle) and *Pongo abelii* (Sumatran orangutan).

The remaining 22 of 31 fragments mapped to unlikely candidates for the Scotia Sea including some freshwater and tropical fishes (*S. fluviatilis, Sinogastromyzon puliensis, Pimelabditus moli, Zoarces elongatus, and Aplocheilichthys spilauchen, Apogon maculatus, G. indica*), as well as different aquaculture species, including *Salmo salar* (Atlantic salmon), *Lethrinus erythracanthus* (orange spotted emperor), and *Hypomesus nipponensis* (Japanese pond smelt). Finally, there was representation from terrestrial species such as *Pongo abelii* (Sumatran orangutan), Aquila *audax* (wedge-tail eagle), *Eumeces schneideri* (Schneider’s skink), and *Embernagra platensis* (pampa finch). These were unexpected results as we utilised a custom database that was focused to marine taxa (MARES – COI gene) and protist and microbial taxa (PR2 – 18S gene).

Upon re-querying these 22 species assigned fragments against NCBI, none matched the species from earlier MALT mapping. Instead, 10 of the 22 fragments showed highest similarity to different bacterial orders. Three re-queried fragments (initially assigned to fishes Z. *elongatus*, *S. fulviatilis* and *Squalomugil nasutus*) had no matches. The fragment originally assigned to the terrestrial species *P. abellii* (18S gene) in sample 2H_4 (12.05 mbsf – 11.3 kya) mapped to multiple tunicate species with 100 % identity, including several salps: *Salpa younti, S. thompsoni, and S. fusiforms*. The original PR2 mapped accession (ABGA01187477.10589.12655_U) was mined from NCBI. Conversely, the fragment assigned to *A. audax* (COI – Accession GBIR1094119 Aquila audax 8961 COIN5P) in sample 4H_2 (28.05 mbsf – 63.9 kya) mapped exclusively to *Homo sapiens* mitochondrial genomes with 100% identity. The *A. audax* barcode COI gene (1494 bp), originally mined from NCBI (2019-04-18), was re-queried on BLAST as a cross-reference on (2025-05-23), and did not match to *A. audax* accession, but did have top 100 matches with *Neoaves*, with just under half of top matches with *Accipitrinae* (hawks & eagles).

2. Synthetic Datasets

**Supplementary Figure 3. Boxplot – Violin plots of F1 scores following Kruskal – Wallis comparisons between taxonomic levels. Post-hoc Dunn’s Test p-values with Benjamini – Hochberg corrections for multiple tests are indicated above corresponding taxonomic groups tested. All treatments (including the IODP dataset and the combined IODP + synthetic dataset with different levels of damage and numbers of input fragments) were considered for these tests.**

2.1 Synthetic Data Damage Analysis

All datasets with *n* = 10 input fragment , regardless of damage level, had one fragment misassigned to a non-vertebrate species, *Elliptio complanata*, a freshwater mussel native to North America. Upon closer inspection, this was the same 50 bp fragment in all samples originating from the *H. antarcticus* 18S rRNA input sequence. When re-queried against NCBI, the sequence matched to Teleostei species (67 of 100 listed 18S rRNA sequences), but not *H. antarcticus* (AF518190.1).

And while there was little variation in sensitivity for *T. scotti* across damage level treatments, the sensitivity of the penguin species’ assignments varied noticeably with different damage treatments (Fig. 4). Finally, for eight species, F1 scores were unexpectedly high for the *n* = 10 dataset for all damage levels (Fig. 4). This increased specificity in the species and genus level assignments accounts for the increased overall performance of our pipeline classifier .

**Figure 4. Heatmap of proportion fragment assignment by input species across all synthetic datasets. Purple indicates high sensitivity (e.g., every input fragment assigned correctly at the species level) while yellow indicates low sensitivity. On x-axis: *n*= number of input fragments, nodeam = 0% deamination damage, lowdeam = 3%, middledeam= 7%, highdeam = 18%, and deam = 100 %. Read counts from MEGAN CE were exported per dataset, and the proportion of fragments assigned was calculated as proportion = assigned at species level/ fragment input value n.**

**Supplementary Figure 4. Heatmap of total fragment at each MALT taxonomic assignment of *Pygoscelis antarcticus* 18S and COI gene input in synthetic datasets. Purple indicates high sensitivity (e.g., every input fragment assigned correctly at species level) while yellow indicates low sensitivity. On x-axis: *n*= number of input fragments, nodeam = 0% deamination damage, lowdeam = 3%, middledeam= 7%, highdeam = 18%, and deam = 100 %. Read counts from MEGAN CE were exported per dataset.**

**Supplementary Figure 5. Heatmap of total fragment at each MALT taxonomic assignment of *Harpagifer antarcticus* 18S and COI gene input in synthetic datasets. Purple indicates high sensitivity (e.g., every input fragment assigned correctly at species level) while yellow indicates low sensitivity. On x-axis: *n*= number of input fragments, nodeam = 0% deamination damage, lowdeam = 3%, middledeam= 7%, highdeam = 18%, and deam = 100 %. Read counts from MEGAN CE were exported per dataset.**

**Supplementary Figure 6. Combined Heatmap of total fragment at each MALT taxonomic assignment of *Harpagifer antarcticus* and *Pygoscelis* 18S and COI gene input in synthetic datasets. Taxonomic assignments were filtered to show fragment assignment that was represented in every synthetic dataset. Labels next to gene names indicate NCBI accession number of gene used to make synthetic fragments. Purple indicates high sensitivity (e.g., every input fragment assigned correctly at species level) while yellow indicates low sensitivity. On x-axis: *n*= number of input fragments, nodeam = 0% deamination damage, lowdeam = 3%, middledeam= 7%, highdeam = 18%, and deam = 100 %. Read counts from MEGAN CE were exported per dataset.**

**Supplementary Figure 7. Heatmap of total fragment at each MALT species level taxonomic assignment for all synthetic datasets. On x-axis: *n*= number of input fragments, nodeam = 0% deamination damage, lowdeam = 3%, middledeam= 7%, highdeam = 18%, and deam = 100 %. Read counts from MEGAN CE were exported per dataset.**

2.2 Statistical Analyses

Dataset Variable Interaction testing

- Kruskal wallis tests of interactions of (rank, damage_treatment, and sample context) with fragment input complexity (*n*) ~ performance metric (precision, sensitivity, F1 score) relationship. Computed in R with …. Package. If interaction values were significant, we incorporated variable as interaction term with generalised linear model. If interaction values were not significant across all inputs *n*, then values were aggregated across that interaction variable, to decrease overfitting in statistical analysis.
- After all Kruskal-Wallis tests, a compiled generalised linear model with interaction terms was run to test …
- Across all synthetic + combined datasets, rank and sample context had significant interactions with fragment input complexity (*n*) ~ performance metric (precision, sensitivity, F1 score). Damage treatment did not have significant differences with Kruskal-Wallis test when other variables were considered, so all further values were aggregated across damage treatment.
- Tested generalised linear model: … so this was formula utilised for all spearman correlation analysis.

Supplementary References:

Andrews, S. (2010). "FastQC: a quality control tool for high throughput sequence data". Available online at: [http://www.bioinformatics.babraham.ac.uk/projects/fastqc/](https://www.bioinformatics.babraham.ac.uk/projects/fastqc/).

Armbrecht, L., Herrando-Pérez, S., Eisenhofer, R., Hallegraeff, G. M., Bolch, C. J. S., & Cooper, A. (2020). An optimized method for the extraction of ancient eukaryote DNA from marine sediments. *Molecular Ecology Resources*, *20*(4), 906–919. <https://doi.org/10.1111/1755-0998.13162>

Arranz, V., Pearman, W. S., Aguirre, J. D., & Liggins, L. (2020). MARES, a replicable pipeline and curated reference database for marine eukaryote metabarcoding. *Scientific Data*, *7*(1), 209. <https://doi.org/10.1038/s41597-020-0549-9>

 Bushnell, B. (2014). “BBMap: A Fast, Accurate, Splice-Aware Aligner.”

Chernov, T. I., Tkhakakhova, A. K., & Kutovaya, O. V. (2015). Assessment of diversity indices for the characterization of the soil prokaryotic community by metagenomic analysis. *Eurasian Soil Science*, *48*(4), 410–415. <https://doi.org/10.1134/S1064229315040031>

Clarke, E. L., Taylor, L. J., Zhao, C., Connell, A., Lee, J.-J., Fett, B., Bushman, F. D., & Bittinger, K. (2019). Sunbeam: An extensible pipeline for analyzing metagenomic sequencing experiments. *Microbiome*, *7*, 46. <https://doi.org/10.1186/s40168-019-0658-x>

Ewels, P., Magnusson, M., Lundin, S., & Käller, M. (2016). MultiQC: Summarize analysis results for multiple tools and samples in a single report. *Bioinformatics (Oxford, England)*, *32*(19), 3047–3048. <https://doi.org/10.1093/bioinformatics/btw354>

Guillou, L., Bachar, D., Audic, S., Bass, D., Berney, C., Bittner, L., Boutte, C., Burgaud, G., De Vargas, C., Decelle, J., Del Campo, J., Dolan, J. R., Dunthorn, M., Edvardsen, B., Holzmann, M., Kooistra, W. H. C. F., Lara, E., Le Bescot, N., Logares, R., … Christen, R. (2012). The Protist Ribosomal Reference database (PR2): A catalog of unicellular eukaryote Small Sub-Unit rRNA sequences with curated taxonomy. *Nucleic Acids Research*, *41*(D1), D597–D604. <https://doi.org/10.1093/nar/gks1160>

Herbig, A., Maixner, F., Bos, K. I., Zink, A., Krause, J., & Huson, D. H. (2016). MALT: Fast alignment and analysis of metagenomic DNA sequence data applied to the Tyrolean Iceman. *bioRxiv*, 050559. <https://doi.org/10.1101/050559>

Hübler, R., Key, F. M., Warinner, C., Bos, K. I., Krause, J., & Herbig, A. (2019). HOPS: Automated detection and authentication of pathogen DNA in archaeological remains. *Genome Biology*, *20*(1), 280. <https://doi.org/10.1186/s13059-019-1903-0>

Huson, D. H., Beier, S., Flade, I., Górska, A., El-Hadidi, M., Mitra, S., Ruscheweyh, H.-J., & Tappu, R. (2016). MEGAN Community Edition—Interactive Exploration and Analysis of Large-Scale Microbiome Sequencing Data. *PLOS Computational Biology*, *12*(6), e1004957. <https://doi.org/10.1371/journal.pcbi.1004957>

Ratnasingham, S., & Hebert, P. D. N. (2007). bold: The Barcode of Life Data System (http://www.barcodinglife.org). *Molecular Ecology Notes*, *7*(3), 355–364. <https://doi.org/10.1111/j.1471-8286.2007.01678.x>

Renaud, G., Hanghøj, K., Willerslev, E., & Orlando, L. (2017). gargammel: A sequence simulator for ancient DNA. *Bioinformatics*, *33*(4), 577–579. <https://doi.org/10.1093/bioinformatics/btw670>

Schubert, M., Ermini, L., Sarkissian, C. D., Jónsson, H., Ginolhac, A., Schaefer, R., Martin, M. D., Fernández, R., Kircher, M., McCue, M., Willerslev, E., & Orlando, L. (2014). Characterization of ancient and modern genomes by SNP detection and phylogenomic and metagenomic analysis using PALEOMIX. *Nature Protocols*, *9*(5), Article 5. <https://doi.org/10.1038/nprot.2014.063>
