# Supplementary material for: How low can you go? Establishing detection limits for rare eukaryotes in Southern Ocean sedimentary ancient DNA
Source: Bioinform Adv. 2026 Jun 5;6(1):vbag113. doi: 10.1093/bioadv/vbag113 (PMC13539354; doi:10.1093/bioadv/vbag113)
Supplement: Supplementary file 1 [file vbag113_supplementary_data.zip › Supplementary Files Description .docx]

Supplementary Files Description

1. Supplementary File 1: All dataset raw read counts

Summary information of collapsed, post –adapter removal and -deduplication for all IODP, synthetic, and IODP + synthetic datasets. Also calculated from taxonomic detection of Eukaryota, Vertebrata, Notheniodei, and Spheniscidae in proportion of total mapped reads to MARES + PR2 custom database.

2.Supplementary File 2: Sensitivity and Precision calculations

Summary of calculated sensitivity and precision for each synthetic and IODP + synthetic datasets, calculated from raw taxa total counts of taxonomic assignment exported from MEGAN CE. Precision and sensitivity were calculated at the species, genus, and family taxonomic levels.

3.Supplementary File 3: F1 scores

F1 scores calculated for all synthetic and combined IODP + synthetic datasets, from sensitivity and precision metrics. All equations can be found in main text Methods 3.1.

4. Supplementary File 4: IODP reanalysis vertebrate assignment metadata

Summary of all Vertebrata taxonomic assignment from IODP site U1538 site reanalysis, with extracted read fragments, as well as metadata from MALT taxonomic assignment.

5.Supplementary File 5: Shannon Diversity Indices

Shannon Diversity Indices calculated for synthetic and combined IODP + synthetic datasets from post MEGANCE total taxonomic assignment counts, using main text Methods 3.2 and Supplementary Material Methods 4.

6. Supplementary File 6: Percent damage tables

Eukaryote sedaDNA damage (%). Details of % eukaryote sedaDNA damage determined by HOPS post MARES – PRR2 alignment, including average (AV) and standard deviation (SD) across IODP, synthetic, and combined IODP + synthetic datasets at different expected deamination levels.

7. Supplementary File 7. Kruskal-Wallis and Linear Mixed Model Aggregation testing

Kruskal - Wallis and Linear Mixed Model Damage Treatment Aggregation Testing synthetic sample metric values to see if aggregation of damage treatment variable was possible across rank, sample Context, and classifier metric type

8. Supplementary File 8. Spearman Rank Correlations

Spearman Rank Correlations calculated across tested rank, classifier metric, sample context, and tested minimum fragment threshold for rare vertebrates. Source indicates Spearman correlation at Local source (comparing monotonic trends between neighbouring testing variables (e.g. threshold values 10, 20, 50) and Global source (find overall trend values across all testing variables in tested set.
